# Mobile-Assisted Virtual Bonding Enables Breast Milk Supply in Critically Ill Mothers With COVID-19: A Reflection on the Feasibility of Telelactation

**DOI:** 10.7759/cureus.13699

**Published:** 2021-03-04

**Authors:** Roya Farhadi, Shahrokh Mehrpisheh, Roy K Philip

**Affiliations:** 1 Pediatrics Infectious Diseases Research Center, Mazandaran University of Medical Sciences, Sari, IRN; 2 Pediatrics, Mazandaran University of Medical Sciences, Sari, IRN; 3 Pediatrics, University Maternity Hospital Limerick (UMHL), Limerick, IRL; 4 Pediatrics, School of Medicine - University of Limerick, Limerick, IRL

**Keywords:** breastfeeding, covid -19, e-health, mobile health, newborn, telehealth, telelactation

## Abstract

The separation of the mother-infant pair during the immediate postpartum period has been shown to impair the initiation and sustenance of breastfeeding. For critically ill mothers with coronavirus disease 2019 (COVID-19), the imposed isolation generates anxiety for their health and that of the offspring. In this study, we present a few cases where a favorable outcome was observed through a telehealth initiative for mothers with severe COVID-19 pneumonia, which involved sharing pictures and videos of newborn infants with the mothers during the ongoing severe acute respiratory syndrome coronavirus 2(SARS-CoV-2) pandemic in a maternity hospital in northern Iran. In addition to the subjective maternal wellbeing offered by the visual and auditory cues from the infant, this technology-assisted telelactation’/‘mobile-lactation’/‘e-lactation’ could potentially enhance the mother's capacity to initiate emotional bonding with the infant and be an adjunct in achieving and maintaining her lactation goals while offering the best nutritional choice for the infant.

## Introduction

Severe acute respiratory syndrome coronavirus 2 (SARS-CoV-2), which causes coronavirus disease 2019 (COVID-19), originated in Wuhan, China in late 2019 and quickly spread around the world and has been declared a pandemic [1]. The first case of COVID-19 in Iran was reported on February 19, 2020, in Qom city, and it spread to all 31 provinces by March 5, 2020 [2]. As Iran faced the first wave of COVID-19 pandemic outside of China, even before the European spread, the healthcare system had to respond rapidly through local initiatives, adaptations, and innovations.

The use of telemedicine and mobile-assisted technology during the COVID-19 pandemic could accelerate clinical service provision while preventing the spread of the virus, and telehealth services can play an important role in the remote management of vulnerable cases [2,3]. It has already been suggested that postnatal virtual visits and remote consultation could support healthy mothers in establishing breastfeeding practices during the pandemic while reducing the number of times mothers and newborn infants need to leave their homes. Mothers with COVID-19 infection were recommended to express breast milk or establish breastfeeding, adhering to the recommended infection prevention measures [4]. The recent report about the detection of anti-COVID-19 antibodies in breast milk further reinforces the value of breast milk for infants during the pandemic [5]. When health authorities restrict or limit parental access to neonatal intensive care units (NICU) for mothers with confirmed COVID-19, telehealth and mobile-health innovations could provide the solution through virtual communication and it could play the role of stress-reliever as well.

During the initial days of the pandemic, our policy was to keep newborn infants in isolation in NICU in cases of confirmed maternal COVID-19. Many mothers were critically ill to express breast milk (EBM), and the physical separation, lack of opportunities for bonding, and absence of skin-to-skin contact with the infants were all contributing to their inability to initiate and continue breastfeeding.

Methods

We hypothesized that in resource-limited settings, commonly used smartphone applications such as WhatsApp (Facebook, Inc., Menlo Park, CA) could be provided to mothers who are kept in isolation due to COVID-19 and could act as an adjunct to facilitate mother-infant bonding and support breastfeeding. A quality improvement project (QIP) initiative sourced mobile smartphone units for each of the separated mother-infant pairs, and the hospital infection prevention and control (IPC) team vetted the procedures for cleaning, aseptic precautions, and safe usage. Local Wi-Fi connections at the hospitals were offered through a secure password-protected system, and the smartphone mobile application WhatsApp with end-to-end encryption was used in our feasibility study. After obtaining parental consent for virtual communication, the head nurse of NICU coordinated the possible e-visiting hours with the isolation unit in the postnatal ward. Two nurses and one attending neonatologist were involved in the project, and four nurses were trained to establish a virtual video connection via the WhatsApp application. Virtual visitation was facilitated anytime between 10 AM to 7 PM (twice during the morning and evening nursing shifts). The intervention consisted of a daily voice message to the mother initiated by the head nurse at the NICU. In addition, mothers had the option to call NICU at their convenience as well. Every morning, recorded pictures and videos of the baby were sent to the mother by a designated nurse and a video call was made at the request of the mother. During the video call, the mother was encouraged to talk with the baby’s clinical team as well as see her baby. At the discharge of mothers, with visiting restrictions to the hospital still in place, mobile-assisted communication was continued via WhatsApp on the mother’s personal phone at home.

In this report, we describe our experience relating to three mother-infant pairs who were offered virtual bonding during the COVID-19 pandemic in northern Iran.

## Case presentation

Case 1

**Figure 1:**
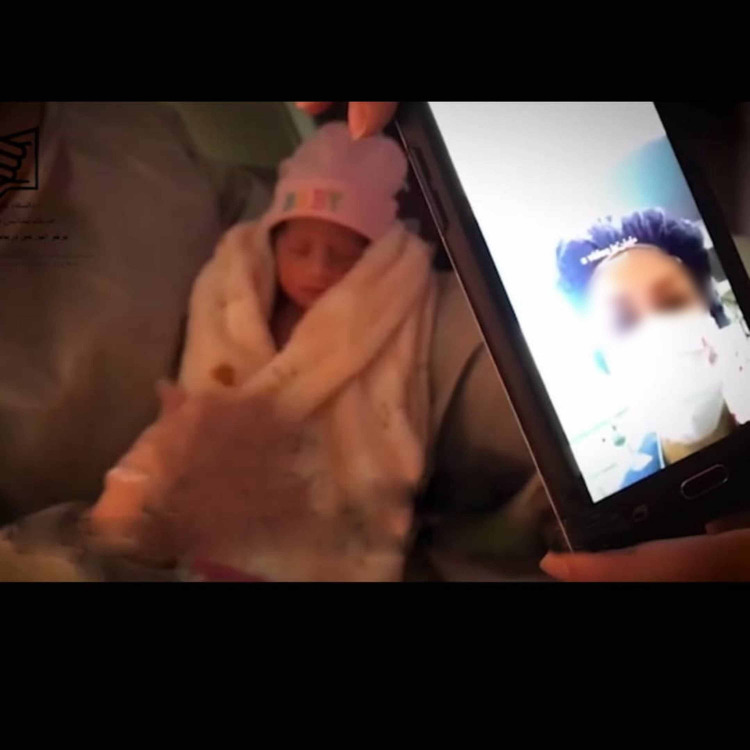
Virtual bonding of mother-infant pair aided by mobile-assisted technology Virtual communication is being established with the help of a nurse. The image of the mother is seen on the mobile screen. Written permission was obtained from parents for publishing this image

A 31-year-old primigravid pregnant woman with severe mitral stenosis developed COVID-19 pneumonia with hypoxia and acute respiratory distress syndrome (ARDS) and was admitted to the cardiac intensive care unit (CICU) at 32 weeks of gestation in late March 2020. The clinical decision was made to deliver the baby and transfer it to NICU in a maternity unit 3.3 kilometers away. The mother required non-invasive ventilatory support and responded well. The infant was initially offered formula feed, and on day three, periodic virtual visits between mother and infant were initiated so that she could see her baby and interact with the nurse. The infant’s nurses shared the pictures and videos of the baby in real-time with her mother (Figure 1).

She was able to receive periodic updates on the infant’s progress, and the visual and auditory cues, as per her own account, “released her emotions and worries”. The infant was confirmed to be COVID-19-negative and the father acted as the courier to bring EBM multiples times a day. The mother persevered on the manual expression of milk till mid-June when she underwent mitral valve replacement surgery. During the virtual periodic follow-up appointments until mid-November, both the mother and baby remained in good health and the baby still received half of the nutritional requirement in the form of breast milk.

Case 2

A 26-year-old gravida two, para one (G2P1) pregnant woman with severe COVID-19 pneumonia and ARDS was admitted to the ICU in Sari, north of Iran, in early April 2020. Emergency cesarean section was performed at 31 weeks of gestation, and a baby girl weighing 1,420 grams was born and admitted to the NICU for nasal continuous positive airway pressure (CPAP) respiratory support and was kept in isolation. Virtual bonding via WhatsApp photos and live videos was commenced on the second day and nurses, during each shift, facilitated the virtual mobile-enabled closeness. They used a phone adapter to fix the device over the incubator by adhering to the IPC guidelines. The infant’s COVID-19 status was proved negative, and even after maternal discharge after day seven, she continued the sessions four times daily. EBM volume increased consistently from the fourth day, and she was transported to the hospital from home by the family members till the discharge of the infant on day 30. Since this was early in the course of the pandemic with limited information available on the suitability of breast milk for infants of COVID-19-positive mothers, it was challenging initially both for the nurses and the mother to decide and persevere with EBM feeding. Reverse transcriptase-polymerase chain reaction (RT-PCR) sample of breast milk was also reported negative, providing additional reassurance. Sixteen days after birth, when the mother could finally visit the NICU following the local IPC guidelines, it was a rewarding moment for her to move from the virtual bonding to the warmth of touch, feel, and cuddle with ease, immediately commencing the skin-to-skin care through Kangaroo mother care (KMC) principles. It was intriguing to observe that the volume of expressed breast milk remained the same regardless of whether the mother’s lactation was supported by virtual contact or directly, even though we initially expected the latter to offer higher volume.* *The infant was discharged home on the third of May fully breastfed, which continued till mid-November.

Case 3

During the second wave of COVID-19 disease in Iran in July 2020, a 36-year-old woman delivered her baby boy weighing 3,200 grams at 36 weeks of gestation following preterm premature rupture of membranes (PPROM) and a confirmed diagnosis of SARS-CoV-2 through RT-PCR. The mother was admitted to the COVID-19 isolation ward and as per the unit policy, the infant was isolated in the NICU. Even though he was reported to be well at birth, the infant became apneic at two hours of age; chest X-ray showed mild parenchymal infiltrates and the infant’s pharyngeal swab RT-PCR for SARS-CoV-2 at 24 hours of life was positive. Non-invasive ventilatory support was required and the infant’s condition steadily improved. Despite mild shortness of breath, the mother was eager to explore EBM collection as she was symptomatic and could not visit the NICU. Being a healthcare professional herself, the mother welcomed the offer of a video connection with the baby, and after seeing baby videos via WhatsApp, she managed to send the first sample of EBM to NICU. The mother was discharged from the hospital on the fourth day after delivery, and she continued to communicate virtually with the baby; her two other children at home quarantine were also able to share the moments and be a part of this communication. After 10 days of virtual communication between the mother and the infant, the mother became asymptomatic, and skin-to-skin contact and breastfeeding were established; on day 14, the infant was discharged. Even when the mother was initially symptomatic with COVID-19, the continuation of WhatsApp calls and real-time videos after discharge from the hospital also might have helped her to sustain an exclusive breast milk feeding, and it is worth reporting that the infant’s investigations had also returned positive for SARS-CoV-2 at 24 hours of age.

Detailed information related to virtual bonding for all three cases is given in Table 1.

**Table 1:** Individualized details of mobile-assisted virtual bonding for the three mother-infant pairs

Intervention characteristics	Case 1	Case 2	Case 3
Time of initiation after delivery (day of life)	3rd	2nd	2nd
Type of intervention (via WhatsApp)	Video call + picture/video sharing	Video call + picture/video sharing	Video call + picture/video sharing
Frequency of virtual visit per day (average)	2-3	3-4	2-3
The average duration of each video call (minutes)	5-10	3-5	3-5
Total days of virtual bonding	20	14	10

## Discussion

Virtual connectivity has received increasing acceptability in the healthcare sector, and it has been sought out by patients and healthcare providers since the implementation of restrictions in response to the COVID-19 pandemic. Telemedicine has mostly been used in the management of adult patients with chronic conditions or for the homecare of children with chronic illnesses [6,7]. The use of telemedicine or video calls in the homecare of newborn infants has also been described [8].

Telehealth strategy allows the mother to access resources that would support breastfeeding, and ‘telelactation’ has already been reported as an efficient and convenient method [9,10]. Telelactation is an innovation that can connect breastfeeding mothers to remotely located lactation consultants through audio-visual technology [11]. Manufacturers of breast pumps have also reported the use of telelactation video visits to breastfeeding mothers through their mobile apps; however, there is limited data in the published literature on this aspect of mobile telelactation, and there is a dearth of evidence to support the use of mobile apps for NICU parents [11,12].

As Iran was the first country impacted by COVID-19 beyond China, the Iranian health authorities initially followed the National Health Commission of China recommendation postponing routine hospital visits of parents of newborns during the COVID-19 pandemic [13]. In addition, it was also recommended to allow video visitation or outpatient consultation as a policy for caring for newborn infants born to mothers with suspected or confirmed COVID-19 [14]. Overall, parents face emotional difficulties when a newborn infant is admitted to NICU, and during the COVID-19 pandemic, parents faced additional challenges as they could not see or interact with their sick or premature infants during the critical window for development and emotional bonding [15].

Yeo et al. carried out an internet-based telemedicine program for NICU using a web camera installed by the baby’s cot, and the family was able to view real-time video images of their infant through a secure portal via cell phone or internet browser. Virtual visitation was well appreciated by parents when sick newborns required care in the NICU. The authors have recommended further evaluation to assess the role of virtual visitation in improving post-discharge transition care of neonates [15].

Huang et al. observed satisfactory outcomes in mild and severe cases of COVID-19 pneumonia based on the use of the online and offline multidisciplinary quarantine observation forms and online monitoring. They demonstrated that the online multidisciplinary self-quarantine model could actively promote the management of adult patients with COVID-19 [16].

Although there have been media reports on telehealth and telemedicine adaptations to facilitate virtual mother-infant bonding, there is insufficient evidence-based literature to support the use of telemedicine technology to allow NICU families to visit their babies during the COVID-19 pandemic [17]. Gaulton et al. introduced a video monitoring system using a small bedside camera at every cot at the Abington Hospital-Jefferson Health’s NICU in the US for families when their infants were admitted during the COVID-19 crisis [17].

To the best of our knowledge, our report is only the second one about the use of telemedicine related to the visiting policy pertaining to newborn infants born to mothers with COVID-19 and the first to suggest its potential application as an adjunct in initiating and sustaining breast milk supply. Although it is a hard decision, different neonatal units have adopted various policies to restrict visiting the NICU during the COVID-19 pandemic based on the evolving guidelines from WHO and national bodies. During this unprecedented time of crisis, to ensure that the baby, parents, and healthcare workers (HCWs) are safe, evidence-based guidelines need updating on a periodic basis. Our experience demonstrates the supplementary role that could be played by a simple initiative such as contact through WhatsApp to foster breast milk extraction support even for critically ill mothers, through periodic virtual visits and live videos enabling virtual bonding, thereby easing the transition to successful breastfeeding as soon as possible. Mothers also remain reassured visually that the HCWs are doing everything they can in the NICU to support their infant’s health and wellbeing. It is worth commenting that all the nurses who took part in this initiative provided excellent feedback (even though some of them had initial apprehensions), and now the Mazandaran regional maternity unit offers a telelactation program to mothers who remain in isolation with infants admitted to NICU. Recent reports about the inhibitory capacity of breast milk against COVID-19 [18] give additional impetus to our approach, which has the potential transferability to resource-limited settings. The previous experience of the unit in achieving induction of lactation in challenging clinical scenarios through international collaborative efforts [19] also provided confidence to our neonatal team to pursue a low-cost mobile phone-enabled e-lactation in the midst of a pandemic.

We acknowledge certain limitations in this report. There were impediments to virtual visitation on occasions due to poor internet connection and unpredictable maternal health issues, limiting the virtual access to infants. The small number of mother-infant pairs in our feasibility study holds inherent shortcomings. Although some valuable experiences like olfactory and tactile senses are not achieved between mother and infant in virtual bonding, the overall success of this program and the positive feedback it received show that this model could provide the basis for further research involving a larger cohort to examine the efficacy of telelactation in the NICU during and beyond the pandemic.

## Conclusions

Virtual mother-infant bonding with mobile-assisted technology helps to connect COVID-19-infected mothers with newborn infants remotely and enables mobile lactation. Techniques offering telelactation could play a supplementary role during the COVID-19 pandemic and potentially hasten the emotional recovery of mothers separated from their infants during the postnatal period by playing an important role in breast milk initiation and sustenance.
